# Scaling-up community-based rehabilitation programs in rural Thailand: the development of a capacity building program

**DOI:** 10.1186/s12913-022-08458-5

**Published:** 2022-08-20

**Authors:** Sirinart Tongsiri

**Affiliations:** Faculty of Medicine, Mahasarakham University, Tambon Talad, Muang, Mahasarakham, 44000 Thailand

**Keywords:** ICF, International Classification of Functioning, Disability and Health, Community-based Rehabilitation, CBR, Persons with disabilities, PWDs, Implementation, Quality of life, Scaling-up framework, Thailand, Disability

## Abstract

**Background:**

Approximately 15% of the world population have some forms of disability and their quality of life is compromised. According to Thailand Persons with Disabilities (PWDs) Empowerment Act, B.E. 2550 (2007), PWDs are entitled for benefits ranging from medical care to social support services**.** The CBR framework and the International Classification of Functioning, Disability and Health (ICF) can be used to promote the interdisciplinary approach across staff from different organizations**.** This study aimed to demonstrate the capacity building strategy for user organizations and resource teams, the key components in environment of scale-up as described in “WHO/ExpandNet Scaling-up Framework” to promote the implementation process of CBR interventions in Thailand.

**Methods:**

The study was conducted with a network of representative from five sub-districts in Thailand. A set of capacity building training courses was designed. Fieldworkers were trained to administer the ICF questionnaire to collect data of PWDs in community. A qualitative interview was conducted to investigate the changes of the interdisciplinary teams.

**Results:**

The total of 1,783 PWDs data were collected during 1 April 2018–30 December 2019. All of them have, at least, one type of impairment and one type of difficulty in activity of daily living (ADL). Needs of assistive devices and home modifications were also recognized. Individual ICF profiles can also developed to monitor change of their functioning after receiving services. After the discussions in the qualitative interviews, it is indicated that their perceptions towards work with PWDs were changed. The six steps in capacity building include: dialogue, team building, disability role-play; ICF data collection and analysis; developing individual care plans for PWDs; home and environmental modifications for PWDs; training to promote employment opportunities; and evaluation of the care plan.

**Conclusions:**

The study highlighted the innovative training methodology for building up the capacity of staff to work as a team and to become agents of change to set up a strategic plan for delivering CBR interventions in their own settings.

## Introduction

### Disability situation

Approximately 15% of people in the world have a disability, and their quality of life (QoL) is compromised [1]. To meet the Sustainable Development Goals (SDGs), persons with disabilities (PWDs) should be prioritized for inclusive development and the recommended implementation programs include: addressing inclusion barriers; mainstreaming disability in the implementation of SDGs; investing monitoring and evaluation of the progress; and strengthening the implementation means, for example, finance, technology, capacity-building and multi-stakeholder partnerships [2].

Two million Thai people are registered as persons with disabilities (PWDs); according to the Persons with Disabilities Empowerment Act, B.E. 2550 (2007), they are entitled to benefits ranging from medical care to social support services [3]. Multiple organizations are responsible for providing interventions and services to enhance the QoL of PWDs.

To guarantee human rights and equal basis of PWDs, the Empowerment of Persons with Disabilities Act, B.E. 2550 (2007), Sect. 20 states the rights of PWDs to access: medical services; education; public facilities; disability allowance; legal assistance; personal assistants; sign language interpreters; home modifications;; vocational training and employment services; and loans without interest [4]. Disability Service Centers (DSCs) have been developed to manage and coordinate with other organizations to provide such services for PWDs [4] and include two types: general DSCs and provincial DSCs. General DSCs are located at the sub-district level and can be established by local government organizations, non-governmental organizations (NGOs) or disability people’s organizations (DPOs) whereas provincial DSCs are the provincial office of the Ministry of Social Development and Human Security. General DSCs mainly provide cares for individual PWDs and provincial DSC is responsible for PWDs data collection and analysis, multidisciplinary collaboration with different organizations [5].

PWDs in Thailand face challenges in accessing services and public spaces [6]. The unemployment rate of PWDs is high; approximately 75% of the PWDs labor force are unemployed [7], despite the law being enforced; one reason is due to the disparity between PWDs and employers’ expectations. Challenges from PWDs’ perspectives include inaccessible workplaces, employer’s limited knowledge, difficulties in commuting from home to the workplace, low wages, and unequal opportunities for career promotions. Challenges from employers’ perspectives included concerned about modifying the environment (e.g., making toilets and ramps accessible for PWDs) and concerns that PWDs would not perform well and become a burden to their workplace [7].

A report produced by the Faculty of Social Administration at Thailand’s Thammasat University stated that although the Thai government has a set of procedures to address the marginalization and exclusion of PWDs (e.g., the registration of PWDs, direct cash transfers to PWDs or caregivers, and loans with no interest in helping business startups), less than 50% of Thai PWDs can benefit from available opportunities [8]. The researchers recommended that, to enhance PWDs’ accessibility to services, key actors should collaborate and communicate with each other, clear procedures should be established on the roles and responsibilities of staff, an effective disability database should be developed, a mechanism should be created to monitor and evaluate programs based on data regarding PWDs’ needs and functions, and support should be increased for community-based services. Several conceptual frameworks are required to achieve these expected outcomes.

### Community-based rehabilitation (CBR)

The World Health Organization (WHO), the International Labour Organization (ILO), and the United Nations Educational, Scientific and Cultural Organization (UNESCO) jointly defined community-based rehabilitation (CBR) as follows: “a strategy within general community development for the rehabilitation, equalization or opportunities and social inclusion of all people with disabilities” [9, 10].

**Fig. 1:**
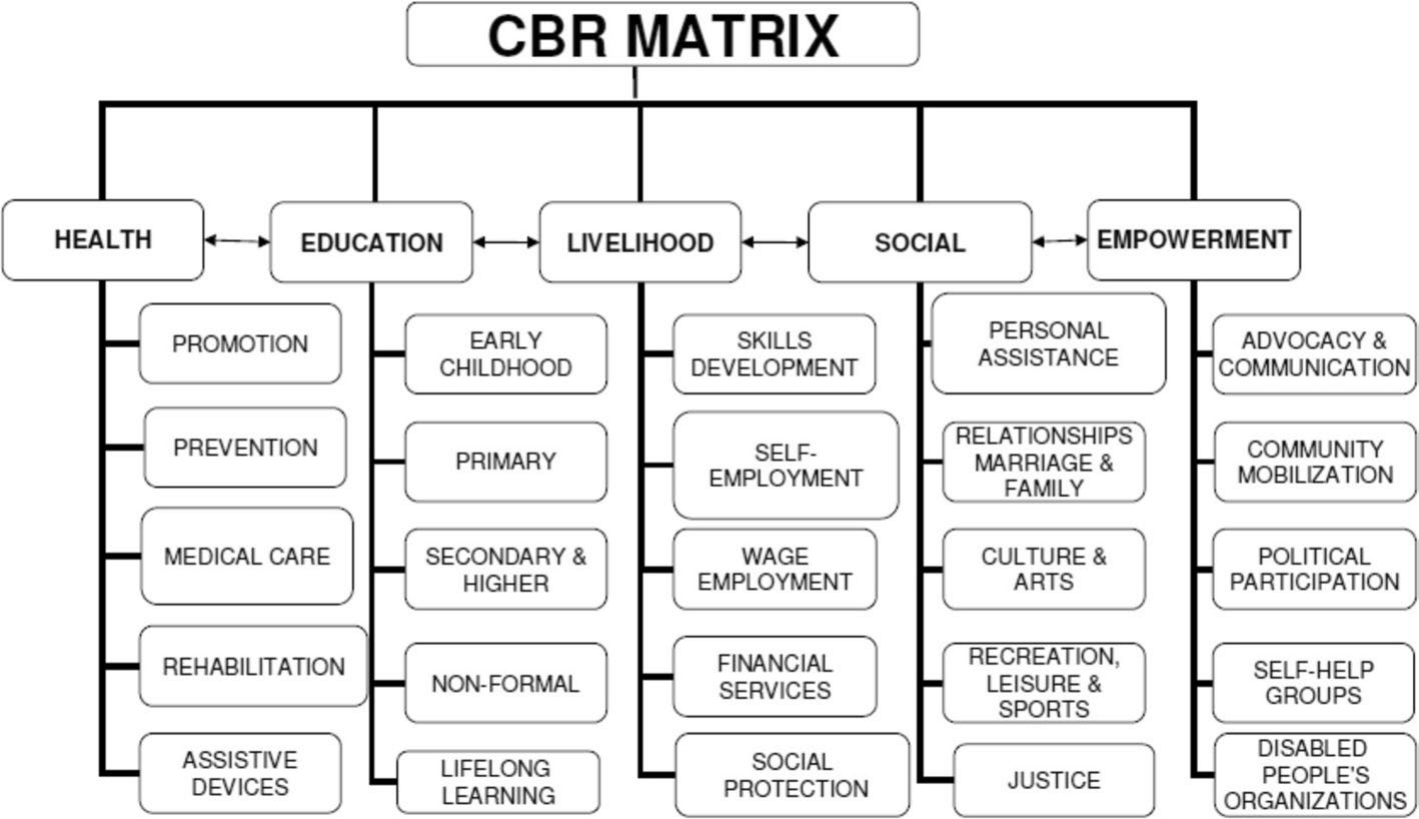
CBR Matrix

The CBR framework was developed from outreach rehabilitation services to involve multiple sectors in the community to provide comprehensive care for PWDs, and eventually became a strategy for community development because the QoL of PWDs involves more than one component. To promote the QoL of PWDs means to call for collaboration among all community sectors. The CBR matrix comprises five dimensions: medical, education, livelihood, social, and empowerment, and are presented in Fig. 1.

Wickenden et al. [11] reported that CBR can be used to realize the UN Convention on the Rights of Persons with Disabilities (UNCRPD) and achieve inclusive development initiatives. They also suggested that a CBR program should involve all community stakeholders, including PWDs, from the beginning of CBR program implementation. The culture and beliefs of the community play significant roles in human capacity building and the design of CBR activities.

CBR programs involve multiple management steps [12, 13]. Most of them primarily involve community resource mobilization, knowledge transfer from service providers to PWDs and families and communities, seamless services among institutions ranging from the community to the national levels, data collection and analysis, and teamwork between medical and social services.

The CBR conceptual frameworks and their comprehensive dimensions can be used to guide the provision of services for PWDs in the community. Several literature reviews have attempted to address the effectiveness and impact of CBR programs [14, 15] and reported that programs have commonly been conducted in low-and middle-income countries in Asia, Africa, and Latin America. There are different designs of CBR programs ranging from visiting PWDs in the community, providing independent living (IL) training for PWDs, and promoting access to education for children with autism. Most of the literature comprises qualitative studies that aim to ensure social inclusion and community resource allocation to promote the QoL of PWDs and their families. Additional quantitative studies should be conducted to estimate the effectiveness and efficiency of programs in which measures are required to address the needs of PWDs and monitor the outcomes of programs.

### International Classification of Functioning, Disability and Health (ICF)

**Fig. 2:**
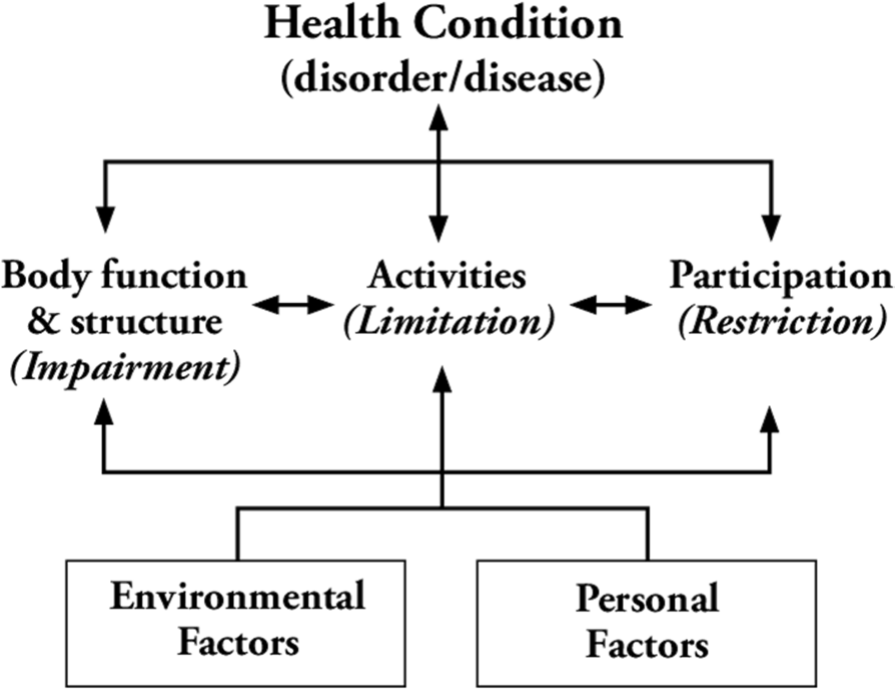
ICF conceptual framework

The International Classification of Functioning, Disability, and Health (ICF) is a classification system that defines an individual’s functioning, which are dynamic interactions between disease, body functioning and body structures, activities, participation, environmental factors, and personal factors [16, 17]. The ICF conceptual framework is illustrated in Fig. 2.

The ICF framework clearly reflects the interconnection between social and medical paradigms and makes it possible to design an interdisciplinary approach for service providers across medical and social services to be able to work together. Without a framework that can harmonize and integrate the working process, conflicts are likely to occur, given that perceptions of disabilities may differ between health and social workers. For instance, the functioning of a person encountered from stroke with left hemiparesis, can be improved not only by him using a single cane to assist his walking capability, but also a handrail for pulling himself up from sitting and employment opportunity from community.

Data collection via the ICF framework is also possible, as it provides codes and qualifiers to identify the limitations of activities and restrictions in participation. A systematic literature review showed that the ICF has been used in both clinical rehabilitation and non-clinical contexts (e.g., employment and disability eligibility) [18, 19]. This allows for the possibility of developing data based on the needs and functioning of PWDs, outcome measurement, and monitoring, leading to organizational performance improvement to enhance the efficiency of programs. ICF can be used as a tool to report functioning outcomes of PWDs after the implementation of CBR interventions. For example, a qualifier of ICF code representing walking (d450) of a person can be changed from 2 (moderate impairment) to 1 (mild impairment) after receiving a single cane (Assistive devices) and participating in a rehabilitation program (Rehabilitation).

### Scale up framework

Rehabilitation for PWDs is a complex task. Implementing a program successfully executed from one area may not guarantee the same level of accomplishment in other areas. WHO/ExpandNet defined scale-up for the health sector as “the effort to magnify the impact of health service innovation successfully tested in pilot or experimental projects, so as to benefit more people and to foster policy and program development on a lasting basis” [20]. It is also suggested that scale-up should be viewed as a learning process to build capacity of personnel in local settings to expand “options, knowledge, processes and technologies such that people build capacities to make better decisions and/or influence decision-making authorities” [20].

**Fig. 3:**
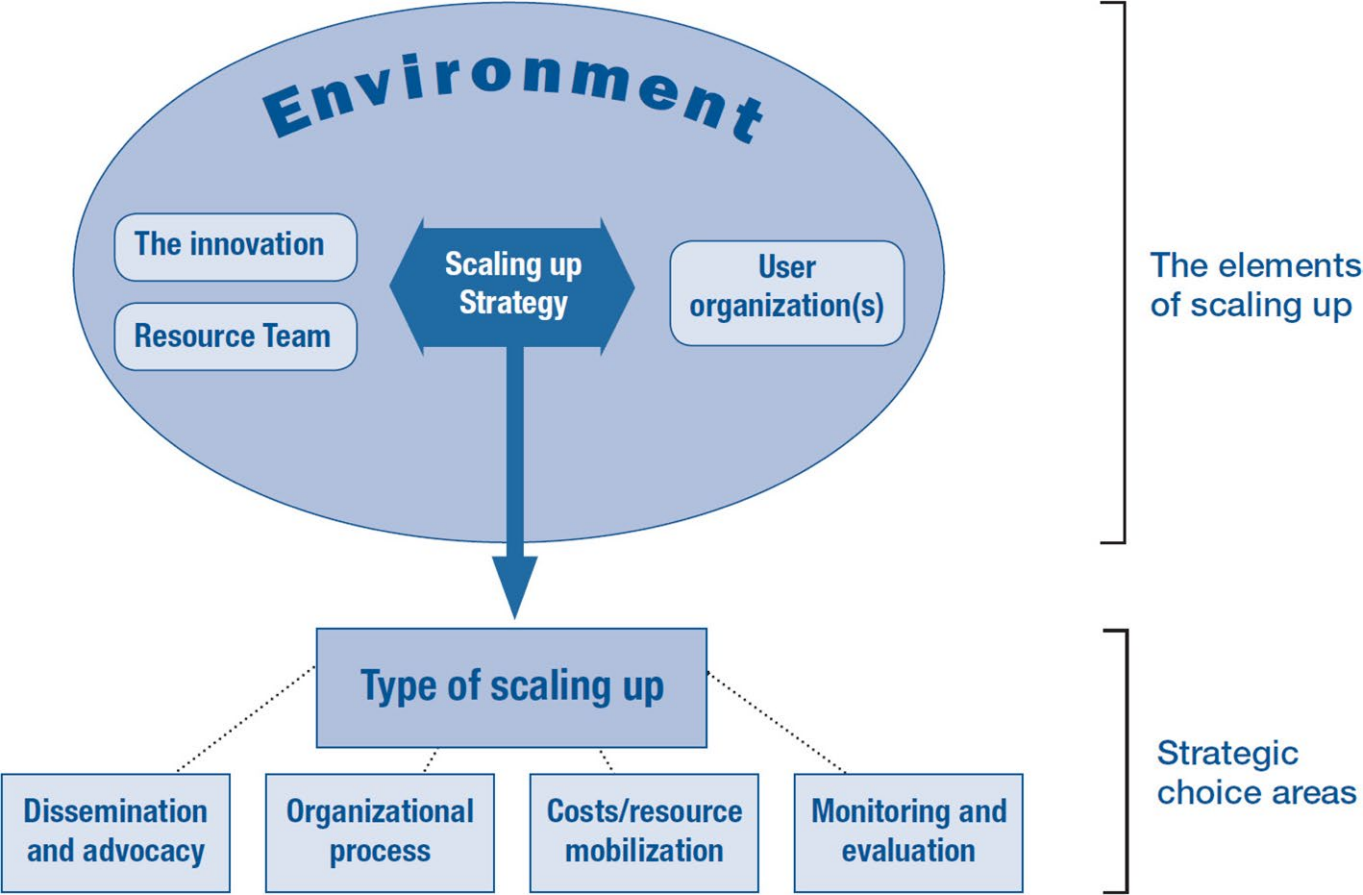
Scaling-up framework

Figure 3 illustrates elements of a scale-up comprise between the innovation, user organization, environment, and resource team and the scaling-up strategy, which can include: dissemination and advocacy; organizational process; costs/resource mobilization; and monitoring and evaluation [21].

Examples of interventions undertaken by the scaling-up framework include: introducing injectable contraception in Vietnam, community-based health planning and services in Ghana, and family planning initiatives in Brazil [22] as well as the integration of a population, health, and environment project in East Africa [23]. In Vietnam, the injectable contraceptive depot-medroxyprogesterone acetate (DMPA) was introduced to improve quality of family planning program at the national level. To scale-up the intervention (DMPA), staff from Ministry of Health as resource teams, provided training programs for user organizations, i.e., staff from health offices, on knowledge and skills in counselling and information to support the use of the method. Situation analysis was also conducted to assess environment of health offices to improve client flow, logistic management and infectious control. It is found that the continuation rate of DMPA became steadily higher and staff’s knowledge of comprehensive use of contraceptive methods and infectious control were increased.

This study aims to demonstrate the strategy to scale-up the CBR interventions in Thailand community settings using the ExpandNet Scale-up framework. CBR interventions are perceived differently among PWDs and care providers from different sectors in the community [24]. To scale-up the CBR interventions (the innovation), capacity building for user organizations and resource teams, both in PWDs functioning data collection and appropriate disability conceptual framework is important. This will allow members from different organizations to gain essential knowledge and skills needed to provide comprehensive services to improve PWDs’ quality of life. R In this study, the interventions to be scaled up include a package of interventions delivered to PWDs living in community settings.

We offer a new approach of innovative training methodology, rather than focusing solely on technical skills, networking (participatory organization development approach), communication, and understanding the difficulties that PWDs experience, which can enable providers, healthcare workers, community leaders, and PWDs to become agents of change.

## Methodology

A research team: one researcher from the Faculty of Medicine, Mahasarakham University, Thailand and one research assistant from the Collective Change Foundation, Thailand, jointly developed the study aiming to develop a scaling-up strategy for CBR intervention. The study was conducted between 2016–2019 and financially supported by the Thai Health Fund. Five districts from five provinces—Mae Hong Son, Chiang Rai, Chiang Mai, Chainat, and Songkla—were requested to participate in the study as “leading districts.” These five districts were selected because they were members who were active and interested in participating in the study.

A team approach was used so that staff from multiple organizations—including nurses, physiotherapists, social workers, community leaders, and local government organization employees— were trained together. Table 1 indicates that the training curriculum included basic principles of disabilities and technical skills in home modification and ICF-based data collection and development of skills and competencies in interpersonal relations, communication, organization development and participatory approaches, focusing on topics of right-based services for PWDs. Disability role play, during which participants were asked to adopt various physical limitations (e.g., by using a wheelchair, axillary crutches, or a cloth tied over their eyes) and perform ADL, encouraged service providers to understand the experience and difficulties from the perspective of PWDs which enabled service providers to appreciate what their job meant to PWDs and their family members. Self-evaluation and reflection were introduced throughout the courses to facilitate feelings of empowerment.

**Table 1:** Training courses and forums

Course	Aims	Content
No.1 Dialogue and Disability Role-play. Action plan development	A network of stakeholders from cross-sectors and the understanding of difficulties encountered by PWDs Action plans of individual sub-districts	A 3-day course featuring 1 day of dialogue and networking, 1 day of disability role play, followed by 1 day of world-café meeting developing comprehensive care plan for PWDs in the sub-districts
No.2 ICF-based data collection	Data collection skills and plan for PWDs data collection analysis in the sub-districts	A 3-day course comprises 1 day of ICF conceptual framework lecture and ICF-based questionnaire, 1 day of data collection training in the field and 1 day of data input and analysis using the ICF program
No. 3 Home and Environment Modification training course	Capacity building in knowledge and skills of home modification and the community resource mobilization	A 3-day course presenting 1 day of Universal Design conceptual framework and the application of ICF in analysis home modification, 1 data training in the field and 1 day of home modification planning in the sub-district
Forums 1–3	Knowledge management across sub-districts from which inspiration can also be generated	A yearly forum (3 forums in total) was arranged to be a platform for managers and stakeholders from 5 sub-districts to share and learn from each other

Quality of life of PWDs has more than one dimension. Functioning of PWDs is the result of the interactions between multiple factors. To understand functioning of PWDs (based on ICF) and realize their needs (based on CBR framework), there must be more than one method used in data collection. The study conducted both quantitative study and qualitative study.

Quantitative study aims to collect functioning data of PWDs. The research team provided materials including an ICF-based questionnaire developed by Tongsiri and Riewpaiboon [25] where 46 ICF codes—4–6 qualifiers representing levels of impairments or difficulties (0 represents no impairment/ difficulty, 1 mild impairment/ difficulty, 2 moderate impairment/ difficulty, 3 severe impairment/disability and 4 total impairment/ disability, 8 for not specified and 9 for not applicable) for each code—were used to measure functioning of PWDs. To ensure that the providers were adequately trained in the workshop,both lecture and hand-on practices were used in the training. A slot of "question and answer" session was available after the workshop to provide clarification of data collection. There also were healthcare providers with rich experiences in ICF data collection to coach and supervise participants in the field. The team also trained participants to conduct data analysis using the Microsoft Excel program to identify needs of PWDs. After completing the training courses, the participants were expected to adopt a leadership role to work with other providers in their setting. The researcher was available for additional training on ground if requested.

The participants had time between the courses allowing them to implement knowledge and execute skills from the training course. The participants were asked to conduct a baseline ICF-based data collection of PWDs in their own community to realize the needs of PWDs. When the ICF data collection of each site was completed, the data were filled in Microsoft Excel spreadsheets and analyzed by the research team. Subsequently, the data were presented to each site’s staff to provide a basis of PWDs’ needs for the development of CBR interventions action plans.

The ExpandNet scale-up framework was used as a guide to develop a strategic plan for PWDs to scale-up CBR interventions to promote QoL of PWDs.

A qualitative interview study was conducted to learn participant insights after completing the project. The total of 25 participants; five from each district of the five provinces, who joined the project from the start were invited and all agreed to be interviewed. Each participant represented individual organizations ranging from hospital, health office, local governmental offices, community leaders and PWD’s families. The researcher conducted the interviews in a group discussion which lasted approximately 3 h. A semi-structured interview aimed to elicit participants’ experiences, feelings, and changes that occurred during the project, and their meanings in relation to their “real world” working experiences and perceptions on disabilities and care provisions to improve PWDs quality of life. Two main interview questions included: “*In comparison with your prior experience, could you please identify the changes that occurred after participating in this project*” and “*In the future, what would you like to put in your CBR action plans and what factors or mechanisms can facilitate the success of your plans.*” Interviews were noted and manually coded by one research assistant and reviewed by the researcher. Thematic analysis was undertaken to look for emerging themes and organized to answer the above questions. Participants were encouraged to make further comments on the findings.

## Results

The results of the study were divided in two parts. The first part includes the quantitative data of PWDs living in all five leading districts. The second part is the qualitative data retrieved from a qualitative interview study conducted with staff from five districts. Table 2 presents the demographic characteristics of participants in a qualitative interview.

**Table 2:** Demographic characteristic of participants in an interview

Total participants	25
Gender	
Male	7
Female	18
Average age (years)	43.3 ± 12
Districts	
Chainat	7
Chiangrai	4
Maehongson	4
Chiangmai	5
Songkla	5
Marital status	
Married	21
Divorced	1
Widow	1
Single	2
Education	
Primary school	3
Secondary school	3
University	19
Organizations	
Primary hospital	10
Primary school	1
Local organization	4
Community organization	2
NGOs	5
DPOs	2
Volunteer	1

Note: DPO-Disabled People Organization

### ICF data from 5 districts

ICF data on 1,783 PWDs from five provinces were collected from April 1, 2018 to December 30, 2019 (Table 3). The PWDs’ characteristics are listed in Table 4. Most of the PWDs (61%) were 60 years or older. Hypertension was the most common underlying health condition.

**Table 3:** Number of PWDs classified by provinces

Provinces	Number
1) Chiangrai	648
2) Songkla	445
3) Chiagmai	215
4) Chainat	396
5) Mae Hong Son	79
**Total**	**1,783**

**Table 4:** Persons with disabilities characteristics (*N* = 1,783)

Characteristic	Value
**Gender**	
Male	864 (48%)
Female	919 (52%)
**Age (years)**	
Mean (SD)	58.24 (24.82)
Range	0.5–109
**Marital status**	
Single	115
Married	199
Divorced	22
Widow	170
Unidentified	1278
**Educational attainment**	
Primary school (≤ 6 years)	304
Secondary school	0
Vocational certificate	9
Not attend school/unidentified	1471
**Underlying diseases**	
Diabetes	92
Hypertension	296
Renal disease	25
Allergy	5
Seizure	6
Heart diseases	44
No underlying disease	188

**Fig. 4:**
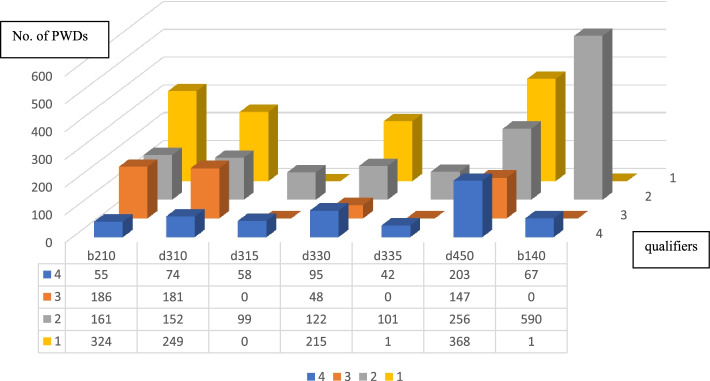
Numbers of persons with multiple levels of impairments Note: b210 Seeing d310 Hearing d315 Communicating with-receiving-nonverbal messages, d330 Speaking, d335 Producing nonverbal messages, d450 Walking, b140 Memory qualifiers: 0-no impairment/ difficulty, 1 -mild impairment/ difficulty, 2-moderate impairment/ difficulty, 3-severe impairment/disability and 4- total impairment/ disability

Figure 4 shows the number of persons with multiple levels of impairment. All participants had at least one type of impairment, including seeing (b210), hearing (d310), communication: receiving (d315), speaking (d330), communication: producing (d335), walking (d450), and memory (b140). Regarding impairment qualifiers, most PWDs had qualifier 0 according to the ICF codes. The greatest proportion of qualifiers—1, 2, and 4—were identified in d450 (walking), whereas qualifier 3 was identified in b210 (seeing).

**Fig. 5:**
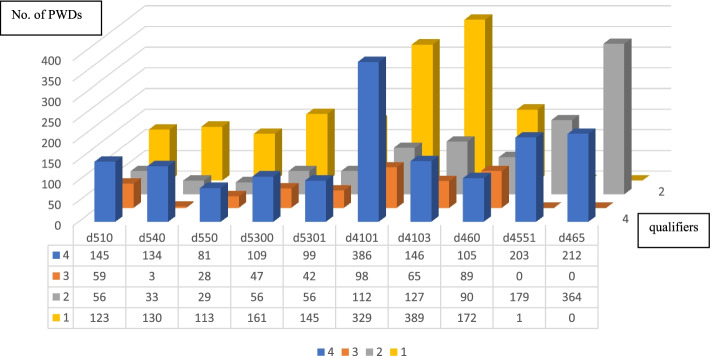
Number of persons with multiple level of disability Note: d510 Washing oneself, d540 Dressing, d550 Eating, d5300 Regulating urination, d5301 Regulating defecation, d4101 Squatting, d4103 Sitting, d460 Moving around in different locations, d4551 Climbing, d465 Moving around using equipment, qualifiers: 0-no impairment/ difficulty, 1 -mild impairment/ difficulty,2-moderate impairment/ difficulty, 3-severe impairment/disability and 4- total impairment/ disability

Regarding activities of daily living (ADL), presented in Fig. 5, squatting (d4101) was the most challenging for PWDs at a severe level (qualifier 4); the second greatest difficulty was in climbing (d4551). The highest number of PWDs with a degree of difficulty at qualifier 3 was identified in squatting (d4101), at qualifier 2 in moving around using equipment (d465), and at qualifier 1 in sitting (d4103).

**Fig. 6:**
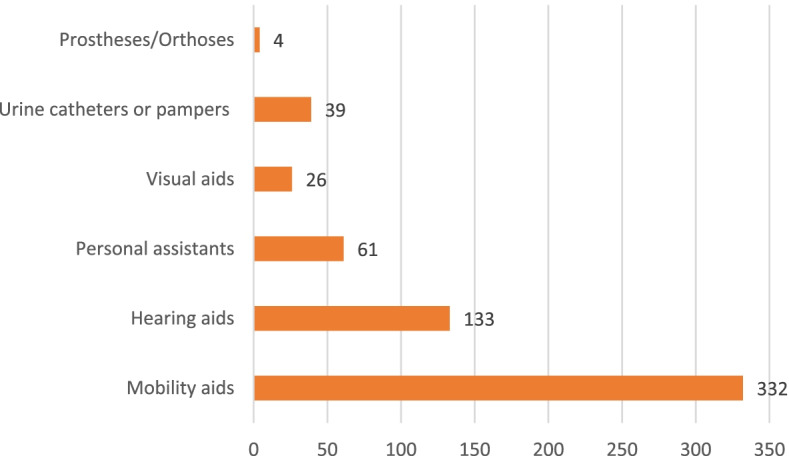
Number and type of equipment required by PWDs

Of the 1,783 PWDs, 332 required mobility aids, 133 needed hearing aids, and 61 required caregivers (Fig. 6). Regarding needs for home and environmental modifications, 194 stated that they wanted toilet modifications, 59 desired modified beds, and 42 wanted ramps. For children with disabilities, five people wanted to go to primary school, four to university, three to high school, and one to secondary school.

**Fig. 7:**
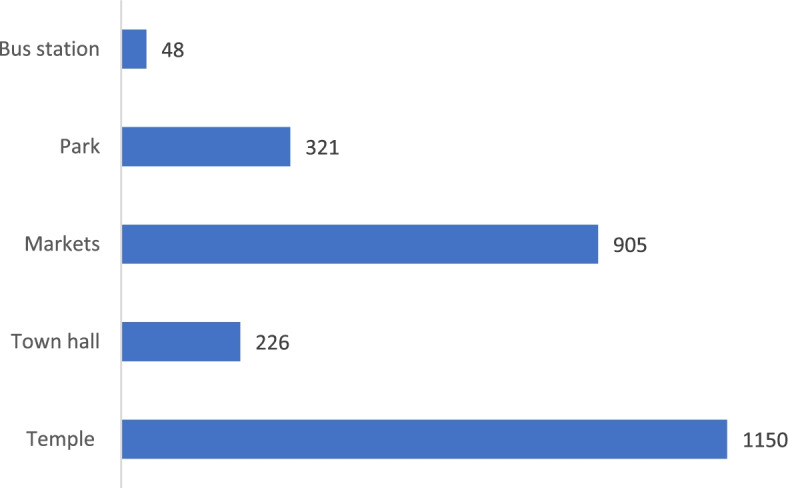
Numbers of PWDs wanted to visit public places

More than 60% expressed that a temple was a place that they wanted to visit the most. Other places included markets, town halls, and parks (Fig. 7).

It is possible that individual ICF profiles can also be tracked down from the data and used to develop individual care plans, as well as to monitor changes in PWDs’ functioning after they received services. After providing all necessary services for all PWDs recruited in this study, we expect to report the comparison of the aggregated 2-point ICF data (before – after) of all PWDs in future study.

After the data collection process was completed, each team leader called for a meeting among the staff to present the results of data collection, identify the needs of PWDs, and develop an annual plan for comprehensive services for PWDs in the community.

**Fig. 8:**
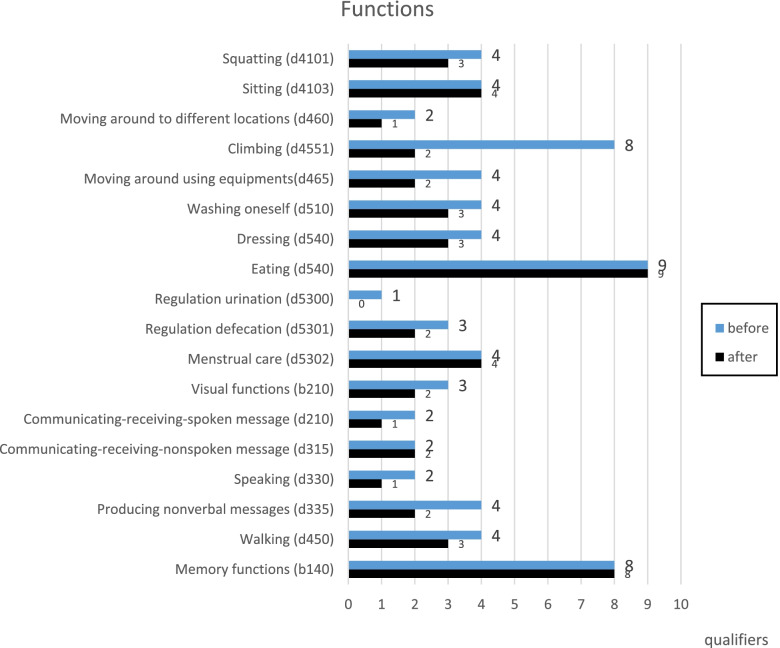
An example of an individual ICF profile

Figure 8 shows the example of the comparison of an individual ICF profile before and after receiving interventions. The improvement of Squatting (d4104), Moving around to different locations (d460), Climbing (d4551), Moving around using equipment (d465), Dressing (d540), Regulating urine (d3300), Regulating defecation (d3301), Visual functions (b210), Communicating-receiving spoken message (d210), Speaking (d330), and Walking (d450) were identified because the qualifiers were moved from more difficult to less difficult after receiving services.

### Changes of disability conceptual framework

After participating in the development of the scale-up process, the unit staff members were invited to share their experiences and the inner changes they sensed had occurred within themselves. Most of the staff, who were service providers, were satisfied with the capacity-building process; this not only increased their knowledge of PWDs’ functioning and helped them understand the difficulties that PWDs face, but also enabled them to expand their networks to involve staff from other organizations. As described in Table 1, Course No.1 (Dialogue and Disability role-play) helped staff from different organizations to become acquainted with others and share their knowledge and experiences regarding working with PWDs. As a result, they realize that they can contribute with each other to provide care for PWDs. Hence, they felt fulfilled and happy to be able to successfully support PWDs and their caregivers. Attempts at developing action plans to promote the well-being of PWDs and the scale-up of the CBR initiative process were addressed. For example, the leader of the Chiang Rai health office said:

“*This work process is not only for PWDs, but I feel happier in working for PWDs. Now, I can understand the experience that PWDs encounter in their lives. I have become a better person because of the training programs. This helps me to see the wholeness of community-based programs; regardless of working with older adults, youth, or PWDs, I can use the same approach. Now, other communities are interested in what we are doing, and I think this working process can be scaled to other areas. We can start by calling for a meeting and sharing our knowledge*.”

Further, experiences in role-play had an impact on service providers’ perceptions of disabilities. A social worker from the Chainat local governmental office said:

*“I have been working with PWDs for a very long time, but this is the first time I have been ‘disabled’ myself. Now I understand their difficulties and I am inspired to empathize with PWDs. I realize the importance of my work to promote their well-being and how this is very significant for them and their families.”*

A government officer from the Chiangrai local governmental office stated:

*“We have to put our hearts into this job. You can work for PWDs anytime, even outside office hours. In role-play, my eyes were totally closed, and I felt extremely uncomfortable. I had to calm myself down and tried my best not to be a burden on my colleagues who were taking care of me. Now, I can understand how blind people feel, and what I, as a government officer, should do to support the blind. It is important to believe in them and to trust their potential.”*

A mother from Songkla Province, who had a child with autism, shared her experience:

*“My son has autism. I cried a lot when my doctor told me [that he had it]. I feel that doctors and nurses have a hard time understanding my feelings. Moreover, finding a school for my son is challenging. I have kept searching for a school for my son, and at the same time, I have pushed the limits to go further to help other parents whose children have autism. Now, we have programs to build the capacity of teachers in schools in our neighborhood. My son can attend school and study with other children. He is very good at drawing and makes me proud.”*

### Future plans and mechanisms for the development of a strategic scaling-up plan of CBR interventions

Dissemination and advocacy of the plans and organizational process and the mobilization of resources from governmental and community organizations are key. To guarantee the sustainability of the interventions after a project is completed, resource mobilization is critical. Participants identified the importance of recruiting additional staff to execute the task:

“*After participating in the project, we realized the burden of staff responsible for taking care of PWDs. We would like to offer them an opportunity to change their mindset towards disability and CBR interventions. They are really tired given the overload of routine works and the imposing of CBR works for PWDs. Capacity building programs is an important starting point. Then they will know that they are not alone in this field, there are many more staff working for the same target group (PWDs), with them they can work with each other and become friends.”*

Additional resources from the community should be mobilized to provide additional capacity building training for staff.

*“We know that home and environment modifications are important factors for improving functioning of PWDs. On top of that, medical care, vocational training and employment should also be provided. At first, we think that the resource for these issues are scarce, but if we can expand our search, we can see other source of funding. For example, the dissemination of funding from Agricultural Fund to the local government organization from which we can apply for funding for conducting the training programs for staff. We can also use money from this funding to buy some assistive devices for PWDs. We realized that many organizations can support us financially, we must look for them and make the access to the funds”*

## Discussion

Quality of life of PWDs is compromised if their needs cannot be addressed systematically and helps cannot be adequately provided. ICF and CBR frameworks are therefore used in this study to realize PWDs needs. The study presented the possibility of using the WHO/ExpandNet Scale-up framework to develop the scaling up strategy for the implementation of CBR interventions in wider community settings. ICF was used as a conceptual framework to enhance insights and understanding of disability among participants from multi-sectors as well as a tool to monitor changes of persons with disabilities after receiving CBR interventions. The study offers a training course that could be used to build capacity of care providers from user organizations and resource teams both from healthcare and social care sectors, in which the areas used in this study can be viewed as a pilot project. Findings from this study are in line with the CBR service implementation steps suggested by Kuipers, Kendall and Hancock [12]. This study also reveals additional insights of staff on changes of their conceptual framework toward persons with disabilities and the scale-up mechanism of CBR interventions.

The training worked well to help change the perspectives of trainees toward PWDs because the participants were asked to “put themselves into PWD’ shoes” to realize the difficulties and experience from PWD’s perspective. This is part of transformative learning where participants encountered with new experiences which can be changed their perspectives towards PWDs. ICF and CBR conceptual frameworks can transform ideas and the way that participants view their work and appreciate the meaning of their work toward PWDs and their family members. Participants can connect with staff from other organizations who have the same goals for PWDs, but who have not yet met in person, so that they can share and work together. In addition to learning technical skills, their perspective on how to view and work in CBR is transformed. This training strategy empowers participants to become “agents of change” in CBR interventions which is similar to the changes of providers in reproductive health services by capacity building educational approach recommended by Diaz and Cabral [22].

Six steps exist in core knowledge to build the capacity of human resources in the community as part of the scale-up strategy to deliver the CBR intention to larger beneficiaries. ICF is key to reshaping the views and enhancing the understanding of staff from different organizations towards PWDs. In this way, complex rehabilitation service tasks can be simplified.

Staff need to be trained in six core areas of knowledge: CBR, ICF, team building, disability role-play, universal design principles, and scale-up framework. The “6 × 6” steps classify the core knowledge into six steps:

- Step 1: Dialog, team building, and disability role-play;
- Step 2: ICF data collection and analysis;
- Step 3: Developing individual care plans for PWDs;
- Step 4: Home and environmental modifications for PWDs according to ICF data;
- Step 5: Training to promote employment opportunities for PWDs;
- Step 6: Evaluation of individual care plans and data collection.

ICF can be used in two ways: 1) Disseminating CBR intervention through the development of a training program to build individuals’ capacity of understanding and working towards functioning aspects, rather than the disability aspect; 2) Monitoring and evaluation: to establish PWDs statistics, needs, and guide the plans for holistic care. ICF can be used for local assessment and environmental analysis before developing the strategic plan for CBR interventions.

Traditional trainings focus on technical skills regarding access to PWDs benefits, for example, home modifications and interest-free loans. This study offers a training methodology composed of the ICF and CBR conceptual frameworks and building a participatory process of decision-making with representation from the community and PWDs. The training package can be offered as a key component of a “scale-up toolkit for CBR interventions” part which is similar to the scaling-up strategy conducted in the implementation of injectable contraception in Viet Nam [21].

A package of CBR intervention is unique because the interventions are complex in that merely one organization cannot provide the services. Multisector organizations, at both national and community levels, are required to get involved. To be able to manage the provision of such a complex intervention, a holistic training methodology is needed to build the capacity of local teams to analyze the reality and limitations of their own communities, thus, empowering them to be agents of change, and to be able to provide holistic care for PWDs.

Team building activities are essential to providing a platform through which members from different organizations can respect each other’s roles and expertise, and increase their self-esteem regarding the value of their work for PWDs. Team practice can be stimulated and they can work together towards their common goals (PWD quality of life).

Our study offers a training package for “lead districts” that want to scale-up CBR intervention to improve the quality of life of PWDs in community settings. Team training is essential to providing a platform for team members to respect each other’s roles and expertise. In addition, team practice could be stimulated as well as the shared aim of working together with common goals.

Involvement of leaders from local governmental organizations, community leaders, healthcare staff from local health centers, social workers, representatives from Disabled Person Organizations (DPOs), and volunteers are an essential part of enabling environments to facilitate the sustainability of the project after the research project ends.

## What’s next?

Other aspects of scale-up still require more attention, including the political commitment and participation of stakeholders, especially at the national level, and financial support throughout the scale-up process. An ongoing support and evaluation team to provide input for the next steps of scale-up types is also crucial. Further study should see the results of quality of life of PWDs after receiving the comprehensive services, application of advanced statistics to interpret and gain additional information of PWDs from the available ICF data.

## Conclusion

ICF and CBR can be used to enhance the understanding and skills of interventions used to improve quality of life of PWDs in community settings. The WHO/ExpandNet Scale-up framework is key to guiding the expansion of the impact of services innovation to benefit a greater proportion of PWDs. This study highlighted the innovative training methodology for building the capacity of staff to work as a team and to become agents of change to be able to set up a strategic scale-up plan for delivering CBR interventions in their own setting.

## Data Availability

The datasets generated and analyzed during the current study are not publicly available due to confidentiality and participants’ privacy reasons but are available from the corresponding author on reasonable request.
